# Efficient Cluster Tree Topology Operation and Routing for IEEE 802.15.4-Based Smart Grid Networks

**DOI:** 10.3390/s23135950

**Published:** 2023-06-27

**Authors:** Jin-Woo Kim, Jaehee Kim, Jaeho Lee

**Affiliations:** 1 Department of Statistics, Duksung Women’s University, Seoul 01369, Republic of Korea; jjin300@duksung.ac.kr (J.-W.K.); jaehee@duksung.ac.kr (J.K.); 2 Department of Software, Duksung Women’s University, Seoul 01369, Republic of Korea

**Keywords:** smart grid, cluster tree topology, IEEE 802.15.4, MAC, routing

## Abstract

Wireless sensor networks (WSNs) have been utilized as communication infrastructure for smart grid applications. The primary requirement of WSNs for smart grid applications is to transmit delay-critical data from smart grid assets ether at the maximum rate or by reducing collision rates. Additionally, WSNs should utilize the limited resources of the network to provide the required long-term QoS. The achievement of these objectives requires a remarkable design of WSN protocols to satisfy the requirements of smart grid applications. In this study, a multi-channel cluster tree protocol is proposed to prevent collisions and increase network performance. In the proposed scheme, the cluster head serves to broadcast a beacon frame containing information on the allocated channels and time slots. This enables the new node to determine its channel and timeslot. A performance analysis reveals that the proposed scheme can achieve a low end-to-end delay and low collision rates compared with the well-known IEEE 802.15.4 MAC protocols widely used in the literature to provide QoS to smart-grid applications.

## 1. Introduction

Globally, climate change has increased the demand for sustainable and environmentally friendly electrical energy. This places a higher strain on the overloaded and outdated power infrastructure predominantly used in most countries [1,2,3,4,5,6]. Meanwhile, the complexity and non-linearity of power distribution networks, coupled with the growing power demand, have led to significant problems known as network bottlenecks. These bottlenecks pose safety risks and challenge the reliability of the power infrastructure [7,8,9,10]. Conventional power grids are vulnerable to communication, monitoring, error detection, and automation issues. As a result, these defects can set off a chain reaction and cause local system failure [11,12,13].

A smart grid is an intelligent power-grid system that improves efficiency by incorporating information and communication technology into the production, transportation, and consumption of electricity. Ensuring the stability of data transmission is crucial because smart grids aim to facilitate real-time information exchange between consumers and suppliers, enabling efficient energy utilization. A smart grid encompasses the entire power grid, from power generation to residential homes, along with associated substructures. By employing two-way digital communication networks, smart grids aim to conserve energy, reduce costs, and enhance the reliability and transparency of power supply to consumers. The network segments that form a smart grid communication structure include the wide area network (WAN), utility local area network (LAN), backhaul, last mile, neighborhood area network (NAN), advanced metering infrastructure (AMI), home area network (HAN), business/building area network (BAN), and industrial area network (IAN). Smart grids should monitor and control power grids in real time for their efficient operation. Therefore, smart grid communication networks have stringent time requirements such as maximum message transmission delay. Smart grid AMI refers to a collection of systems used to evaluate data related to the use of various utility resources such as electricity, gas, and water. It uses load control and demand response to reduce the peak demand on the system and enables dynamic charging to induce energy consumption and cost reduction [14].

An AMI is a system that integrates smart meters to regularly measure energy usage and collect data for analysis. It plays a crucial role in smart grids. To establish this system, a two-way communication network called an AMI network is necessary. The existing AMI network technologies consist of cable-based power-line communication (PLC) technologies. However, owing to their high installation costs and other complexities, electronic methods have become obsolete. The AMI wireless communication structure is a wireless communication method used in the BAN and HAN (called a wireless personal area network (WPAN)). The IEEE 802.15.4 standard is the most likely communication method for processing data through smart grid AMIs. This is because its evaluation is considerably better than that by other AMI communication methods, except in terms of cost [15,16,17,18].

The IEEE 802.15.4 standard features low-speed, low-power, and low-cost chipsets, specifically designed for standardizing short-range wireless communication [15]. Many wireless sensor devices utilize the IEEE 802.15.4 wireless technology because it aligns with the requirements of wireless networks. Smart grids are also well-suited for adopting the IEEE 802.15.4 standard as a wireless technology due to the intercommunication among numerous wireless sensor devices [19,20,21].

The reliability of wireless networks in smart grids directly affects the efficiency of power-grid operation. Therefore, to design an effective MAC protocol for smart grids, the first important consideration is energy efficiency. Because it is difficult for sensor nodes to replace or recharge batteries, extending the life of networks composed of sensor nodes has become a critical concern in smart grids. Other important issues are the network size, node density, and scalability owing to variations in topology. Over time, several factors originate that modify the network topology. Good MACs should accommodate these modifications. Considering these requirements, the IEEE 802.15.4 standard is receiving attention, which is of continuous interest.

The sensor node of a wireless sensor network (WSN) interlocks with a server which is connected to the wired Internet backbone network through a gateway. At this time, the traffic type of the WSN is divided into downstream from the gateway to the sensor node and upstream from the sensor node to the gateway. For downstream traffic, data loss can be prevented even during congestion if the gateway has a sufficient buffer. Typically, a gateway is a device connected to the wired Internet backbone network, located in an area with a stable power supply and a buffer of sufficient capacity. However, because sensor nodes are battery-based low-power devices, these are implemented to minimize the operation of the radio interface and buffer capacity. Therefore, in the case of upstream routers, if the nodes do not transmit data rapidly, data loss may occur at the nodes functioning as intermediate routers in the event of traffic congestion.

In WSNs, it is common to utilize a low-power radio interface. Additionally, the actual traffic generated in WSNs is typically minimal, resulting in the network operating at a low duty cycle. This means that the network remains in a sleep state, where the radio frequency (RF) is turned off most of the time and periodically wakes up to communicate and reduce power consumption. The IEEE 802.15.4 standard is a technology that operates in the ISM band, similar to the IEEE 802.11 standard. However, it faces a challenge in ensuring stable data transmission, due to interference from various pieces of equipment sharing the same band. Moreover, when WSNs are applied in smart factories, congestion points can occur as traffic is aggregated towards the gateway due to the traffic characteristics. To mitigate this, traffic processing measures are required to reduce transmission delays, especially when multiple hops are involved.

Considering this, a plan is required to increase the transmission performance of the network and prevent data loss by operating as a multi-channel while satisfying the requirements of a WSN operating at a low power. Therefore, this study proposes a multi-channel MAC protocol to improve network performance in a smart grid environment. The proposed technique can reduce the routing paths by a new address allocation scheme and transmit data by preventing interference by a multi-channel management scheme.

## 2. Overview of IEEE 802.15.4 Standard

IEEE 802.15.4 is one of the standards for low-rate wireless personal networks (LR-WPANs). It defines the physical and MAC layers. The standard is widely used in communication based on its low price, low power, and low transmission rates. It enables devices to communicate at small distances without additional infrastructure.

A LR-WPAN has the following characteristics [19]:

- Data transmission rates of 250, 100, 40, and 20 kbps;
- Operates in a star or peer-to-peer manner;
- Assigns an address with 16-bit or extended 64-bit length;
- Guaranteed time slots (GTSs) may be allocated;
- The channel is accessed using the carrier-sense multiple access with the collision avoidance (CSMA/CA) mechanism;
- Low power consumption;
- It has 16, 30, and 3 channels in the 2.4 GHz, 915 MHz, and 868 MHz bands, respectively.

In the IEEE 802.15.4 standard, the devices are divided into full-function devices (FFDs) and reduced-function devices (RFDs). An FFD is a device that operates as a coordinator or device. An RFD can only communicate with the FFD as a device that can perform a highly simple function.

The standard optionally provides non-beacon and beacon modes, depending on how the devices communicate. In the non-beacon mode, the devices communicate freely using the unslotted CSMA/CA method without synchronization. In contrast, the beacon mode maintains synchronization between devices based on the beacon frames transmitted periodically from the coordinator and communicates using the slotted CSMA/CA method.

**Figure 1:**
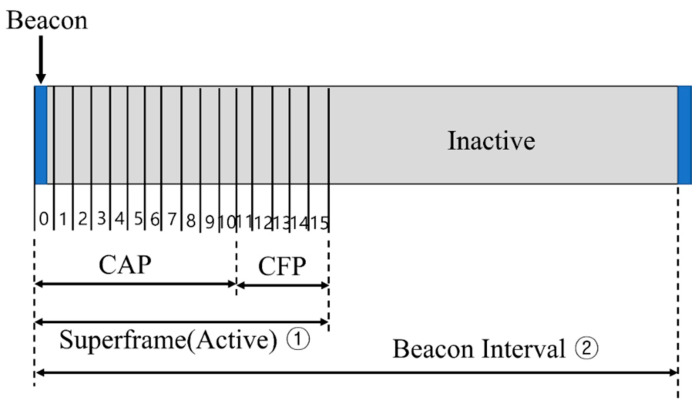
The structure of a superframe.

In the IEEE 802.15.4 beacon mode, the interval at which the coordinator broadcasts the beacon frame is called a superframe. It is divided into 16 slots of equal size. Superframes can be divided into active and inactive durations. Figure 1 shows the structure of a superframe.

The devices can calculate the active and inactive sections using the superframe order (*SO*) and beacon frame order (*BO*) values contained in the beacon frame that are transmitted periodically by the coordinator. The equations for calculating the active and inactive intervals are as follows [15]:

(1) $$Beacon\;Interval(BI)=aBaseSuperframeDuration\times 2^{BO}$$

(2) $$Superframe\;Duration(SD)=aBaseSuperframeDuration\times 2^{SO}$$

In the IEEE 802.15.4 standard [15], the value of aBaseSuperframeDuration in the above two equations is set to 960 slot times, and the ranges of the *BO* and *SO* are limited to 0–14. Furthermore, the values of the *SO* and *BO* should be selected appropriately by considering the relationship between the energy efficiency and transmission delay.

**Figure 2:**
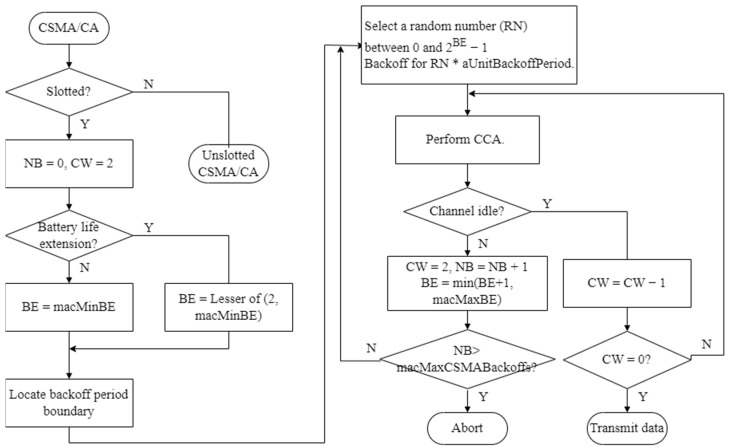
Slotted CSMA/CA mechanism.

In the beacon mode, the devices communicate using a slotted CSMA/CA scheme. Figure 2 shows the operation flow of the slotted CSMA/CA.

NB is a number that indicates how many backoffs the CSMA/CA algorithm requires while attempting the current transmission. It is initialized to zero before each transmission is attempted. The content window (CW) defines a backoff period during which no channel activity should occur before transmission begins. The backoff exponent (BE) is the number of times the device waits for a backoff period before attempting to access the channel. As shown in Figure 2, the NB and CW values are initialized after checking whether the slot is slotted or unslotted. In slotted CSMA, the node waits at the boundary of the backoff period, delays for a random backoff time, and then performs clear channel assessment (CCA) at the backoff period boundary. If the channel is idle, the CW value is decremented by one, and the same process is repeated until CW reaches zero. If the channel is not idle (i.e., the channel is in use), the NB and BE values are incremented by one, within the limit of macMaxBE, as long as the channel is not idle. If the NB value is less than or equal to macMaxCSMABackoff, the random backoff process is performed again. If the NB value is greater than macMaxCSMABackoff, the channel access fails, and the process terminates. The CSMA/CA algorithm is used before transmitting data or MAC command frames within the contention access period (CAP). Otherwise, frames are immediately transmitted following the ACK of a data request command.

Compared with the beacon mode, the non-beacon mode has a highly simple step for performing the operation and causes a negligible transmission delay. However, it has the disadvantage of high energy consumption because it does not have sleep technology that can save communication energy. In contrast, the beacon mode is a suitable method for sensor networks in terms of energy consumption. This is because it can reduce the energy consumption through an active section where the device can communicate and an inactive section where the device can save energy by switching off the communication module. However, because this beacon mode cannot transmit data during sleep intervals, there is a delay in data transmission.

## 3. Related Works

The use of WSNs in general monitoring applications has been discussed widely in the literature [21,22,23,24]. However, only a few publications apply WSNs to smart grid monitoring applications, where the traffic intensity is high and the time delay is a critical issue. For example, the provision of QoS for smart grid monitoring applications was studied in [24,25,26,27]. In [12], the authors proposed the addition of QoS to IEEE 802.15.4, which provides differentiated services for traffic with different priorities in the MAC layer. In [25], the authors proposed modifying the clear channel assessment (CCA) performed by the sensor node to provide service differentiation for high-priority traffic.

In [27], the authors presented an adaptive QoS scheme for WSNs to provide service differentiation and reduce the latency of critical data in smart grid applications. In [21], the authors proposed an algorithm for adaptively assigning a single GTS to nodes with important data. However, a single GTS may not be sufficient to reduce delays in smart-grid monitoring applications with high traffic intensity.

In [28,29,30,31], the authors discussed channel access techniques for multi-hop WSNs. These studies discussed the scheduling of cluster-tree topologies. Most of this work assumes that all the sensor nodes in the network use one-channel access technology to improve network performance. In [31], the authors proposed a multi-channel cluster tree algorithm to prevent beacon frame collisions and GTS collisions between multiple clusters. When the traffic is concentrated, these tree topology algorithms cause bottlenecks in the root and adjacent nodes and increase the latency. In addition, there is a disadvantage in that the routing path is prolonged when nodes at a distance from the root node transmit data between these.

The use of WSNs to support QoS for smart-grid applications has been studied. For example, a method for using WSNs for monitoring purposes in smart-grid applications was studied in [1,32,33]. The challenges and opportunities of using WSNs in smart grid applications have been discussed extensively in [1]. In [32], the authors applied wireless multimedia sensors for monitoring purposes in smart-grid environments. The use of WSNs to reduce and control household electricity consumption was proposed in [33,34]. The authors of [34] proposed a time-of-use (TOU) recognition energy management scheme to reduce the peak load of smart-grid applications based on WSN.

The performance of WSNs in smart grid environments has been described in detail in several studies. For example, the authors in [35] studied various efficient routing schemes of WSNs to provide QoS in smart grid applications. The authors discussed potential areas in which WSNs could be deployed for efficient operational monitoring and control of smart grid applications such as electric transport, distributed energy resources, and storage. In [36], the authors proposed a fiber-wireless sensor network (Fi-WSN) gateway that can prioritize packets in smart grid environments and support QoS for fibe-to-x (FTTX) users. In [37], the authors proposed an adaptive QoS (AQoS) scheme and adaptive guaranteed time-slot (AGTS) allocation scheme for IEEE 802.15.4-based WSNs used in high-traffic intensive smart-grid monitoring applications.

In addition, cross-layer protocols for WSNs have been studied. In [38], the authors proposed the cross-layer protocol (XLP) for efficient communication in WSNs. It provides media access, routing, and congestion control. The authors studied the throughput, latency, Goodfoot, and routing failure rates of the XLP for existing cross-layer protocols in WSNs. The authors of [39] also developed an XLP protocol that combines the physical, MAC, routing, and transport layer functions into an integrated communication framework. The XLP was compared with current state-of-the-art cross-layer protocols for WSNs. In [40], the authors incorporated MAC and routing layer features to route packets from one hop to the next based on weighted progress, considering energy efficiency. A cross-layer optimization solution for power control in the physical layer and congestion control in the transport layer was proposed in [41,42]. In [43], the authors incorporated routing, MAC, and link layer optimization for an efficient design of WSN frameworks. In [44], a scheduling and cross-layer congestion control algorithm for WSNs was proposed. However, this study focused only on two layers: the data link and transport layer.

The QoS is generally defined as an objective measure of the services provided by a network in terms of the bandwidth, delay, reliability, and jitter. That is, the QoS aims to deliver high-priority packets to their destinations with high reliability and low latency. Several QoS-enabled MAC and routing protocols have been proposed. This paper focuses only on a few QoS parameters such as delay [45] and reliability [46]. The authors of [47] proposed a mechanism to reduce the number of CCAs required to forward high-priority packets to the destination of an event monitoring network. Certain studies focused on reducing the duration of CCAs (rather than the number of CCAs) to reduce the end-to-end delay and add priority to certain packets on WSNs, such as the figures proposed in [48]. The effect of CCAs on the performance of energy-constrained wireless networks was discussed in [49,50,51].

One of the most well-known routing algorithms in WSNs is Low-Energy Adaptive Clustering Hierarchy (LEACH) protocol [52]. The LEACH protocol suffers from the issue of uneven distribution of cluster heads (CHs) due to their random selection, resulting in link failures and orphan node problems. To address this problem, the authors proposed a waiting time-based CH selection scheme in [53]. This scheme extends the network lifetime by balancing the load and evenly distributing energy consumption. In [54], the authors proposed an efficient distributed clustering and gradient-based routing protocol (EDCGRP). In [55], the CHs were selected based on their distance to the base station, distance to local neighbors, and remaining energy. In [56], the authors proposed a fuzzy logic-based clustering protocol. This scheme predicts the residual energy to evenly distribute the workload of sensor nodes and improve the network lifetime. In [57], the authors proposed a method for optimal selection of CHs in the grid. In [57], the sensing area was divided into cluster grids, and the CHs were selected based on their distance from the base station and remaining energy. Cluster-based routing protocols have the advantage of low cost for establishing routing paths. However, in ad-hoc communication, the routing paths are limited to the parent node and its child nodes, making the routing path unique and non-optimized.

Advanced power grid systems require strong communication technologies for data exchange between grid devices [58]. The primary purpose of deploying these networks is to enable utilities to facilitate automatic meter reading and obtain highly granular periodic data. These data can be used to provide demand response programs. These systems require highly reliable communication between the head end and metering units [58]. Therefore, in [59], the authors proposed an algorithm to form a multi-hop wireless sensor mesh network for extensive coverage data communication. In [59], the authors proposed an IEEE 802.15.4-based multi-hop mesh network for wide coverage, improved power efficiency, and improved scalability and robust conflict-free networks to ensure the QoS and real-time control of smart grid components.

However, these mesh networks have the problem of increased energy consumption to maintain the links. However, these shortcomings may be offset by other advantages. Meanwhile, because mesh networks use ISM bands, these are vulnerable to interference from various devices that use the surrounding ISM bands. Therefore, in this study, a multi-channel cluster tree topology is proposed to prevent interference and conflict in smart grid applications. The proposed technique has a cluster tree structure. However, it can reduce routing routes using a new address allocation scheme and prevent congestion through alternative routes.

## 4. Proposed Scheme

**Figure 3:**
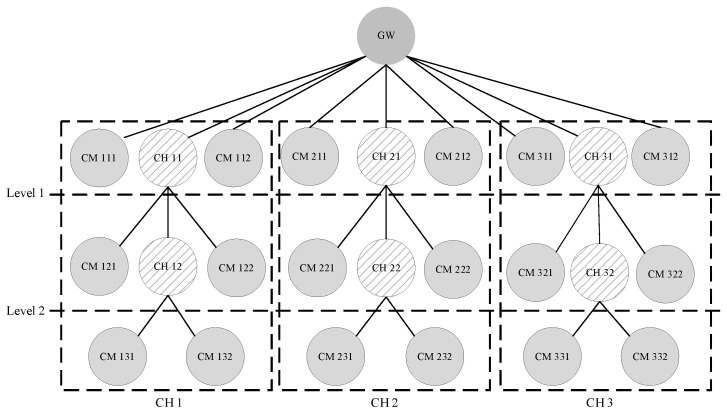
An example of the proposed cluster tree structure.

Figure 3 shows an example of the cluster tree structure used in the proposed scheme.

The IEEE 802.15.4 standard is standardized to support 16 channels in the 2.45 GHz band and 10 channels in the 915 MHz band. While configuring a personal area network (PAN), when a coordinator selects a channel and periodically broadcasts a beacon frame through the channel, devices that receive the beacon frame join the PAN.

These PANs can be extended to a cluster–tree structure, as illustrated in Figure 3. That is, there may be a plurality of PANs in which different channels are selected in overlapping propagation regions. In Figure 3, all the nodes have a single radio interface; GW (FFD) refers to a gateway, CH (FFD) refers to the coordinator of a lower PAN, and CM (FFD or RFD) refers to a member device belonging to a cluster.

In addition, the top PAN coordinators connected to the gateway use channels different from those of the adjacent top PAN coordinators. This forms a cluster tree for each channel. For a multi-channel operation, the gateway operates by changing channels in the order [CH1 → CH2 → CH3], according to the schedule.

### 4.1. The Proposed Superframe Structure

**Figure 4:**
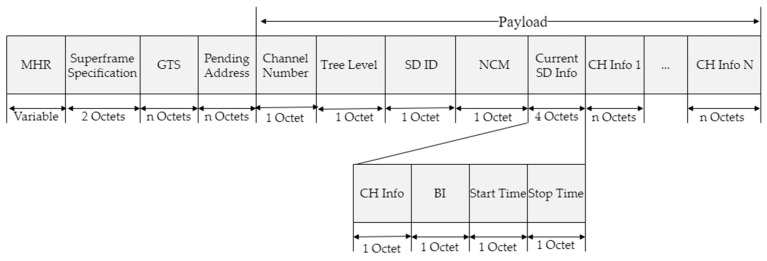
The proposed beacon frame format.

Figure 4 shows the structure of the proposed beacon frame.

In the proposed scheme, the beacon frame is generated and broadcast by the gateway or cluster head. Moreover, a user-defined payload field contains the superframe schedule information of the current channel as well as the superframe schedule information of the other channels. Therefore, when traffic is congested in the current channel, the data frames can be transmitted using the superframe schedule information of another channel (the secondary channel).

In Figure 4, the Channel Number field refers to the number of wireless channels currently used by the network, and the Tree Level field refers to the number of hops from the gateway where the cluster head that sends the beacon frame is located. The SD ID field represents the SD number used by the current cluster head. The Number of Cluster Member (NCM) field represents the number of devices that can participate in the current cluster. This field contains the information used for permit control to ensure load balance and cluster performance while configuring a cluster. In addition, the Current SD Info field stores the superframe schedule information of the cluster head that sends the beacon frame and includes the channel information of the current superframe, beacon interval (BI), and end time (stop time). The CH Info N fields include a bit string that stores information on the SD numbers occupied by the cluster heads in each radio channel. The number of CH Info N fields is determined by the value of the Channel Number field.

**Figure 5:**
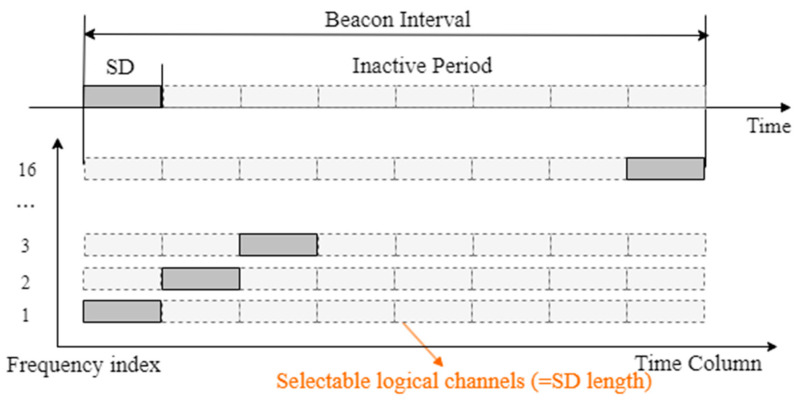
Superframe structure with BO = 6 and SO = 3.

In the proposed scheme, it is assumed that all the clusters use identical BO and SO values. The BI and SD satisfy Equations (1) and (2). Figure 5 shows the structure of the superframe when BO = 6 and SO = 3.

As shown in Figure 5, the divided rectangle becomes a logical channel. The proposed protocol can secure the required number of logical channels regardless of the limited number of physical channels by physically dividing the channels and dividing the SD within the superframe. In the proposed protocol, the channel to which each cluster head is allocated is that to which the concept of logical channel division is applied simultaneously with the physical channel division. As shown in Figure 5, in the case of BO = 6 and SO = 3, it could be divided into eight SDs in the BI. In this case, the length of the bit string of the CH Info N field is 8 bits. In the environment shown in Figure 5, the bit string of each CH Info N field can be expressed as in Table 1.

Each bit constituting the CH Info N field was set to one for the bit pointing to the SD of the channel used by each cluster head. The other bits corresponding to the SD were set to zero. When forming clusters, the gateway selects the SD for each channel, as shown in Figure 5, and records its own bit sequence. The reason for the gateway to select SD for each channel is to communicate with the child cluster heads operating on different channels. The cluster heads joining as child nodes of the gateway choose different channels and select SD that does not overlap with the SD chosen by the gateway. For simplicity of the algorithm, the next SD number of the pre-allocated SD is generally chosen. Information about the pre-allocated SD can be obtained through the CH Info N field included in the received beacon frames. Using the Table 1 as an example, if a cluster head joining the gateway selects channel 4, it checks the CH Info N field received from the gateway. If the next bit of the chosen SD number by the gateway is 0, it selects that SD. In this case, the CH Info N field of the cluster head changes only the 4th bit sequence of the gateway’s CH Info N field, from (00010000) to (0001**1**000).

### 4.2. Proposed Cluster Configuration Scheme

In the proposed scheme, the principles considered by the device when joining a cluster are as follows.

First, the device is joined as close as feasible to the gateway. That is, the node joins the side with a small tree level to reduce the network latency and number of hops. Second, the nodes join by balancing loads such that these do not exceed the maximum number of permissible subscription devices. Third, the node joins as a cluster member if channel and SD allocation cannot be made, even if it intends to join the cluster head.

**Figure 6:**
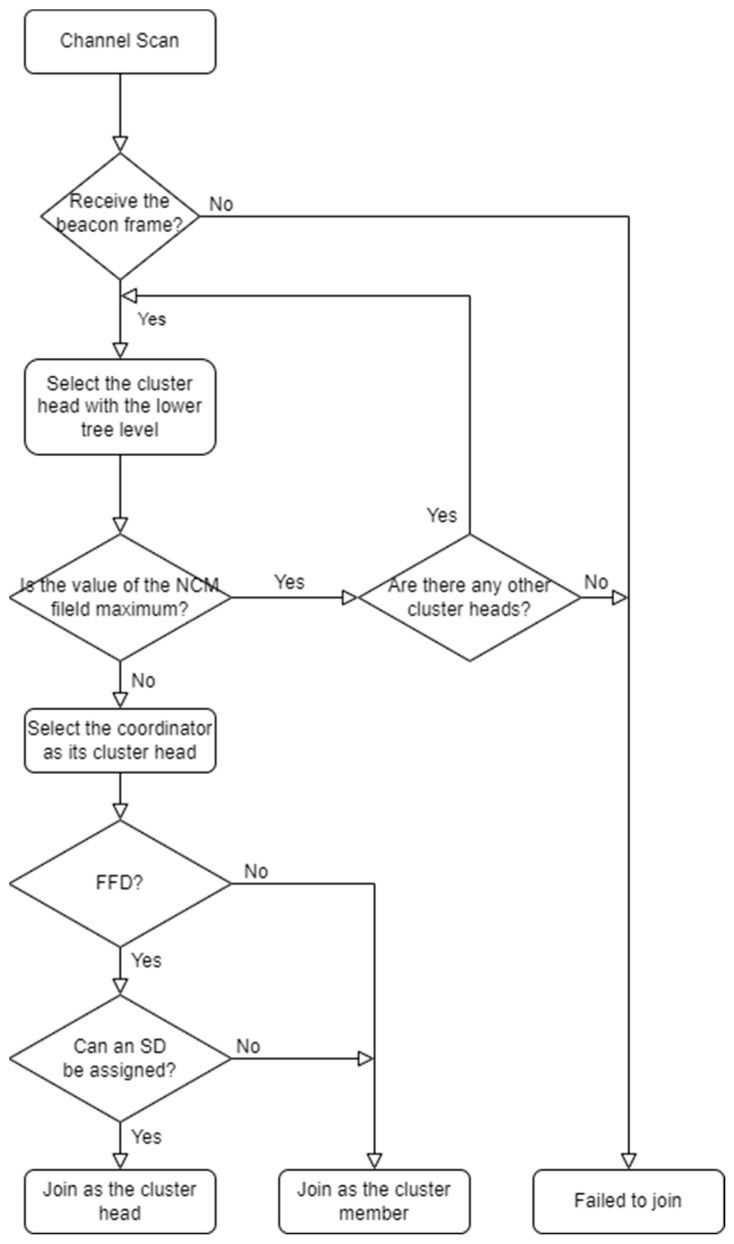
Proposed cluster join process.

Figure 6 shows the cluster-joining procedure for the device in the proposed scheme.

In Figure 6, a node that intends to join the cluster performs the scan process. In this case, a beacon list is obtained using the beacon frames transmitted from the surrounding cluster heads. If no beacon is received, the node cannot join the network. Nodes located close to the gateway receive a beacon frame from the gateway. Therefore, these can join the gateway (to the extent feasible). If the value of the NCM field of the beacon frame received from the gateway does not exceed the maximum value, the SD selection algorithm is executed. In this case, the cluster head becomes the gateway. When assigned its SD, it joins the gateway as a cluster head. Otherwise, other nearby beacon frames need to be searched for. If none are present, it becomes an orphan node. When another beacon frame is received, it joins the cluster head by performing the following procedure.

The node that intends to join the cluster has the smallest value of the tree-level field among the other beacon frames observed. If the value of the NCM field does not exceed the maximum value, the coordinator is selected as the cluster head. Thereafter, the CH Info field is examined to select the SD that did not overlap. If SD allocation is not feasible, the procedure is repeated for the other cluster heads (except for the corresponding cluster head). If an SD is assigned, the node joins the selected parent cluster head as a child cluster head. Otherwise, these join as cluster members.

Nodes that intend to join as cluster members also receive beacon frames from nearby cluster heads through a scan procedure. These then select and join a cluster head with as small of a tree-level field value and an NCM field value as possible as the parent cluster head. If no accessible cluster head is identified, the node becomes an orphan node. The tree configuration process continues until the tree covers all nodes within the cluster.

### 4.3. Proposed SD Selection Scheme

**Figure 7:**
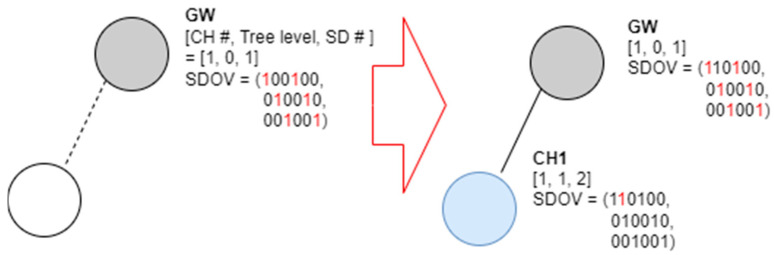
Example of resource selection for the first node joining the gateway in the proposed scheme.

Initially, the gateway displays the SD slot it occupies and the SD slot of the one-hop neighboring node in the CH Info # field and broadcasts a beacon frame. It is assumed that three channels are used and that the number of allocatable SDs is six. It is also assumed that the maximum value of the NCM field of the gateway is set to six and that of the NCM field of the other cluster heads is set to two. In this case, the gateway preempts SD numbers 1 and 3 in advance on channel 1, SD numbers 2 and 4 on channel 2, and SD numbers 3 and 6 on channel 3 in preparation for connecting the 6 cluster heads. Figure 7 shows an example of the proposed SD selection algorithm that operates in a network using three channels.

**Figure 8:**
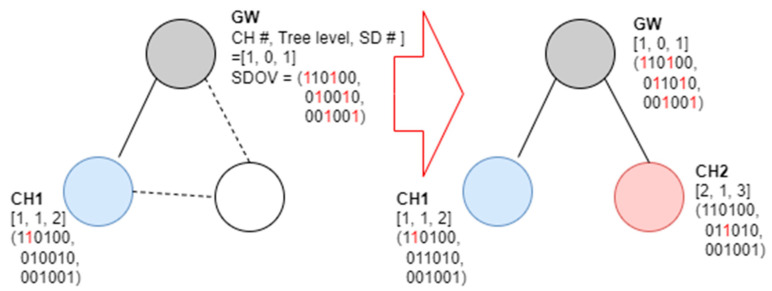
Second example of the proposed SD allocation algorithm.

In Figure 7, the tree level of the gateway is zero, the channel number is one, and the SD number is set to one. If a random node transmits a beacon frame when connected, it examines the beacon frame, selects wireless channel No. 1 and SD No. 2, and functions as a cluster head. Figure 8 shows an example of a new node joining a network following Figure 7.

**Figure 9:**
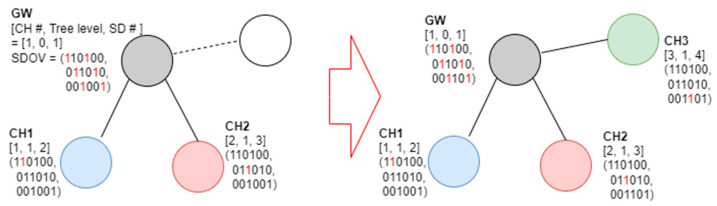
Third example of the proposed SD allocation algorithm.

As shown in Figure 8, when a new node is connected to a network, it may receive a beacon frame from the gateway and CH1. An examination of the received beacon frame reveals that the SD numbers available for channel 1 are 3, 5, and 6; those for channel 2 are 1, 3, 4, and 6; and those for channel 3 are 1, 2, 4, and 5. Therefore, the new node selects channels 2 and 3 to connect to the gateway and operates as a cluster head in the corresponding resource. Figure 9 shows an example of a third node joining a gateway.

**Figure 10:**
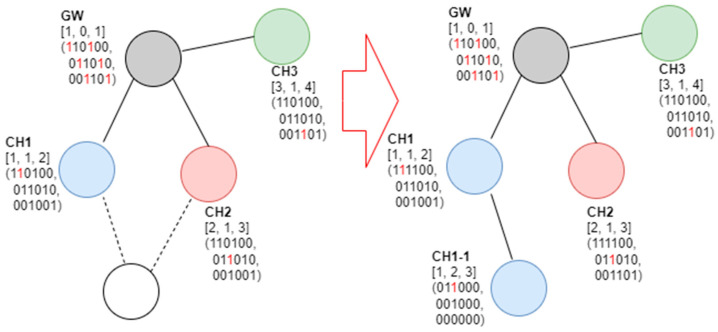
Fourth example of the proposed SD allocation algorithm.

In Figure 9, it can be observed that, if the new node receives the beacon frame from the gateway and CH2, the channels that can be selected through the beacon frame received are numbers 2 and 3. The node can observe that the available SD numbers are 1, 4, and 6 on channel 2 and 1, 2, and 4.5 on channel 3. Accordingly, the node selects channel 3 with a large number of available SD numbers and reports it as CH2. Figure 10 shows an example of a new node joining CH1 as a cluster member.

**Figure 11:**
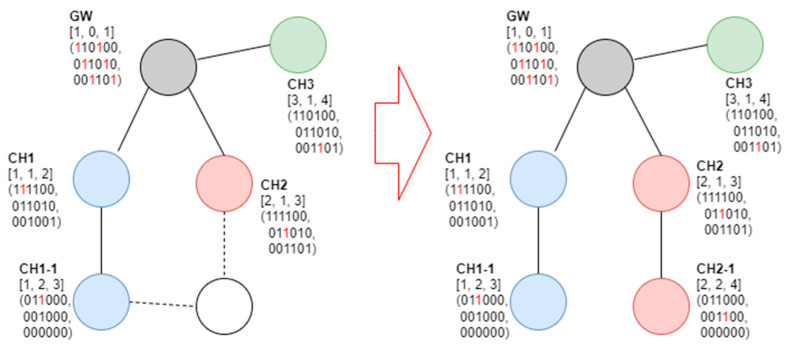
Fifth example of the proposed SD allocation algorithm.

In Figure 10, if the new node receives the beacon frame from CH1 and CH2, the channels that can be selected through the received beacon frame are numbers 1 and 2. It can be observed that SDs 1, 4, and 6 can be used in channel 2 and that SDs 3, 5, and 6 can be used in channel 1. In this case, because the number of available SDs is identical, the selection of the parent cluster does not matter. Here, CH1-2 selected CH1. Figure 11 shows an example of a new node joining CH2.

**Figure 12:**
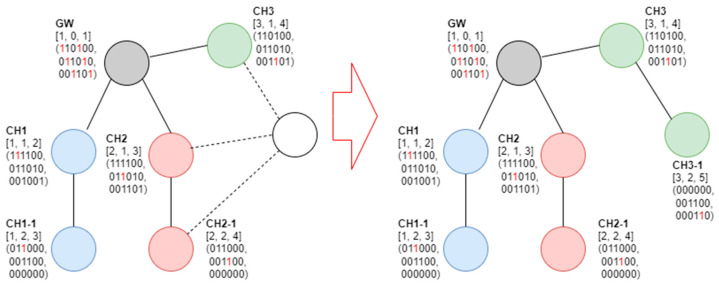
Sixth example of the proposed SD allocation algorithm.

Figure 11 shows that, when the new node receives the beacon frame from CH1-1 and CH2, the configurable channels through the received beacon frame are numbers 1 and 2. In addition, because the tree level of CH2 is 1 and that of CH1-1 is 2, the node selects CH2 (with a small tree-level value) as the parent cluster head. This would then be referred to as CH1-1. Figure 12 shows an example of a new node joining CH3.

Figure 12 shows that, if the new node receives beacon frames from CH2, CH3, and CH2-1, it has configurable channels 2 and 3. The SDs that can be used in channel 2 are numbers 1 and 6, and those that can be used in channel 3 are numbers 1, 2, and 5. Accordingly, channel 3 (with more available SDs) was selected. This fact was noted for CH2 and CH2-1 (i.e., the fifth example of the proposed SD allocation algorithm).

### 4.4. Proposed Network Operation Process

**Figure 13:**
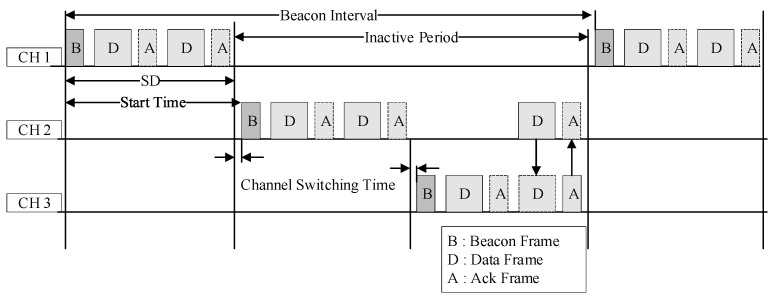
Example of proposed multi-channel operation method using a superframe.

Figure 13 shows an example of the multi-channel operation of the proposed scheme.

As shown in Figure 13, the gateway repeatedly broadcasts a beacon frame for each channel. In this case, each channel comprises a different PAN. Subsequently, the gateway receives the superframe during the activation period through the main channel CH1. If an emergency data transmission occurs in the deactivation section of the main channel, the emergency data can be received using the superframe of the auxiliary channel through auxiliary channel CH2.

As shown in Figure 3, three cluster trees are constructed around the gateway using different channels. The superframe schedule information for each cluster tree is different. Each tree is adjacent to the other trees. If a CH21 coordinator using CH2 needs to transmit emergency data in the current inactive section after transmitting data in the superframe section, it does not wait until the next superframe section and rather transmits the emergency data in accordance with the activation section. Thus, the data can be transmitted rapidly and efficiently without waiting for the next activation period.

**Figure 14:**
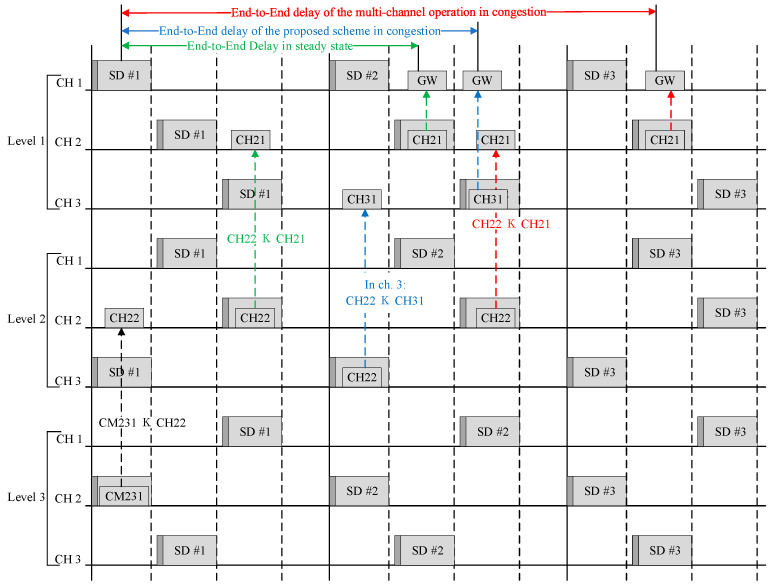
Proposed multi-channel operation scheme.

Figure 14 shows an example of the proposed multichannel operation method.

As shown in Figure 14, each cluster transmits data using the CSMA/CA mechanism in its SD section. However, if data have not been transmitted during the SD period or if they need to be transmitted urgently, this wakes up the activation interval of another auxiliary channel and transmits data. This reduces the latency and prevents data loss.

First, considering the multi-hop transmission delay time during a normal delivery, CM221 (the third-level node of the CH2 channel) transmits data to CH22 (the cluster head) in its activation section. Thereafter, CH22 (a cluster member at the second level of the CH2 channel) transmits data to CH21 (the cluster head) during its activation period. Subsequently, CH21 (a cluster member in the first level of the CH2 channel) transmits data to the gateway during its activation period.

Next, considering the multi-hop transmission delay during data congestion, CM231 (a third-level cluster member of the CH2 channel) transmits data to CH22 (a cluster head) in its activation section. Then, if CH22 (as a cluster member of the second level of the CH2 channel) fails to transmit data to its initial activation section, these are transmitted to its cluster head CH21. Subsequently, CH21 (as a cluster member of the first level of the CH2 channel) transmits data to the gateway during its activation period.

Next, considering the multi-hop transmission delay time when using the auxiliary channel in the proposed technique, CM231 (a third-level cluster member of the CH2 channel) transmits data to its cluster head CH22 in the activation section. If CH22 (a cluster member of the second level of the CH2 channel) fails to transmit data to its initial activation section, the CH3 channel is used as an auxiliary channel to transmit data to CH31 (the first node of the CH3 channel). Thereafter, the first node of the CH3 channel (CH31) transmits data to the gateway during the activation period. Accordingly, the transmission delay time is shorter when an auxiliary channel is used in the multi-channel method during data congestion.

### 4.5. Proposed Addressing Scheme and Routing Scheme

**Figure 15:**
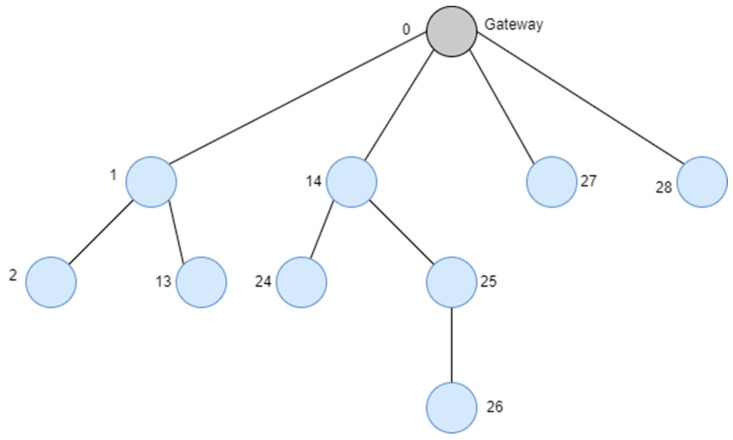
An example for the cluster tree topology in the IEEE 802.15.4 standard.

The tree routing algorithm is a method of transmitting data using distributed address allocation techniques and simple calculations, without the need for separate routing tables. However, it has the drawback of only allowing data transmission between parent and child nodes. Figure 15 illustrates an example of address allocation when each node can have a maximum of four child nodes, a maximum of two routers as children, and the maximum depth of the network is three.

In Figure 15, assuming data is transmitted from Node 2 to Node 26, the routing path follows the sequence 2 → 1 → 0 → 14 → 24 → 14 → 25 → 26. The router with address 14 only remembers the address values of 24 and 25, so it cannot find the sensor node with address 26 that is held by Router 25. As a result, unnecessary hop counts occur due to the drawback of having to descend to the lowest-level nodes. This approach has the disadvantage of frequent collisions as the network size increases.

**Figure 16:**
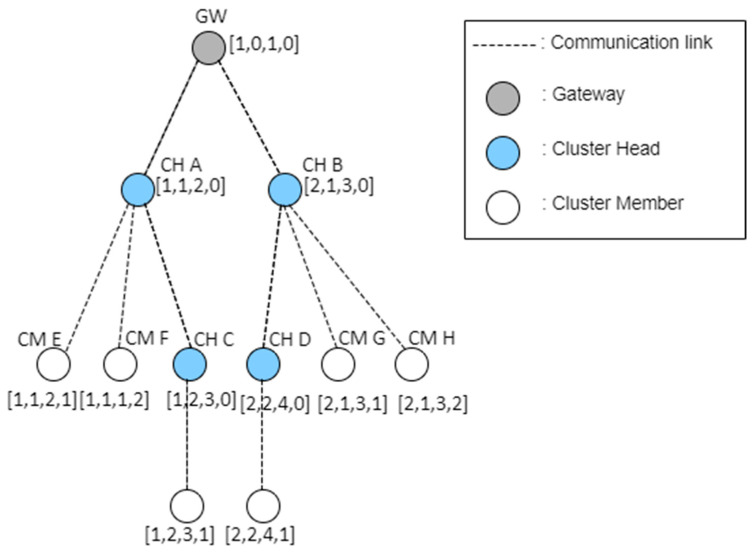
An example of the allocated addresses of devices in the proposed network.

Figure 16 shows an example of an address assigned to a device in the network by applying the proposed protocol.

The proposed address system consists of [channel number, tree level, SD number, and subnet ID]. In this case, it is assumed that the gateway uses channel 1. The tree level of the gateway is set to zero, and the SD number is set to one. The subnet ID of the gateway and all cluster heads are set to zero. The cluster members connected to the cluster head use the channel number, tree level, and SD number of their parent node, as they are, and are assigned a separate subnet ID.

As shown in Figure 16, the tree level of the cluster header is determined to be equal to the number of hops based on the gateway. The cluster head CH A, which first requests the gateway to join the network, selects radio channel 1. The tree level is set to one, which is increased by one in the gateway. The SD is set to two after the SD of the gateway. The subnet ID of the CHA is set to zero because it is a cluster head. When CH B requests network access to the gateway, it selects channel 2 and sets the tree level to 1, similar to the CHA. The standard deviation (SD) is three. CM E and CM F are cluster members of CH A. These are assigned an equal channel number, tree level, and SD number as the parent node CH A and different subnet IDs. CH C uses an equal number of channels as the parent node CH A. However, the tree level is set to two, which is increased by one, and the SD number is set to three, which does not overlap. The proposed scheme modifies the address allocation method. This enables new nodes to be addressed by more intuitive and simple calculations and routing in a simple manner.

**Figure 17:**
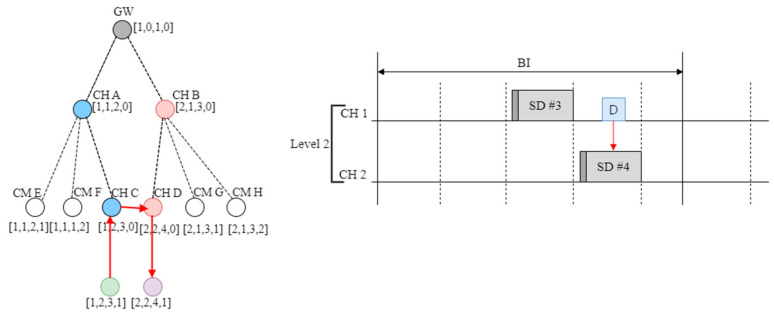
An example of the proposed routing protocol.

Figure 17 shows an example of routing using the proposed address allocation technique. In Figure 17, it is assumed that the address of the origin node is [1–3] and that of the destination node is [2, 2, 4, 1].

As shown in Figure 17, if a packet is to be transmitted from node [1–3] to node [2, 2, 4, 1], node [1–3] transmits data to its cluster head [1–3]. Upon receiving the data, the [1, 2, 3, 0] router examines the destination address, shifts to the SD of the channel, and scans the beacon frame because it is at an equal tree level. When a beacon frame is received, data are transmitted to the cluster head [2, 2, 4, 0]. When the cluster head receives the data, it transmits the data to the node [2, 2, 4, 1], which is its cluster member. If beacon reception is infeasible in the [2, 2, 4] section, the data are transmitted to the cluster head with a section address as close as feasible to Channel 2. The proposed routing technique can more conveniently determine whether the current cluster head is related to the cluster head to which the target node belongs. This reduces the complexity of the routing path calculation. In addition, even when the parent node is different, the channel information of the corresponding cluster head can be determined. Thereby, the data transmission path can be reduced. This proposed technique has a cluster tree structure. However, it can transmit data directly between cluster heads in the communication range. This simplifies the routing path and reduces the energy and transmission time required for data transmission.

### 4.6. Proposed Collision Avoidance Scheme

**Figure 18:**
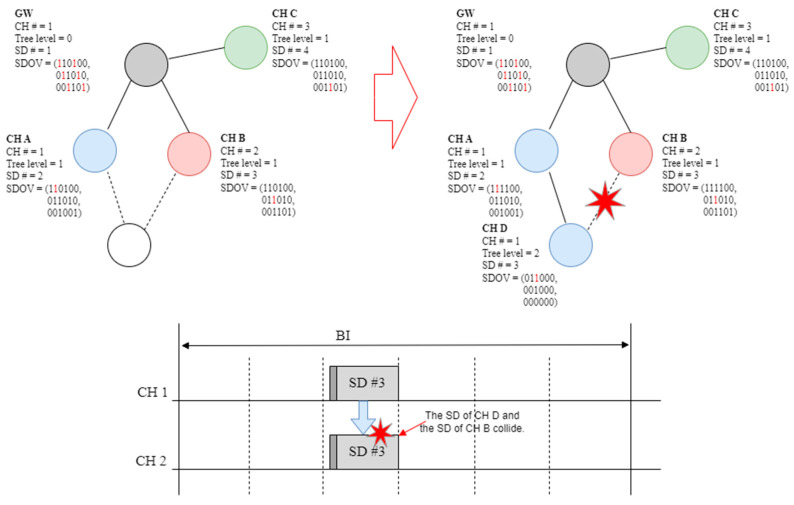
Example of SD conflict when using a secondary channel.

For the multi-channel algorithm described in Section 4.3, there is no means to supplement the interference in the channel of the parent node. Therefore, this section proposes a structure that can simultaneously access the parent node using the main and auxiliary channels to transmit data, even when a problem occurs with the main channel. Figure 18 shows an example of an SD collision using a secondary channel.

**Figure 19:**
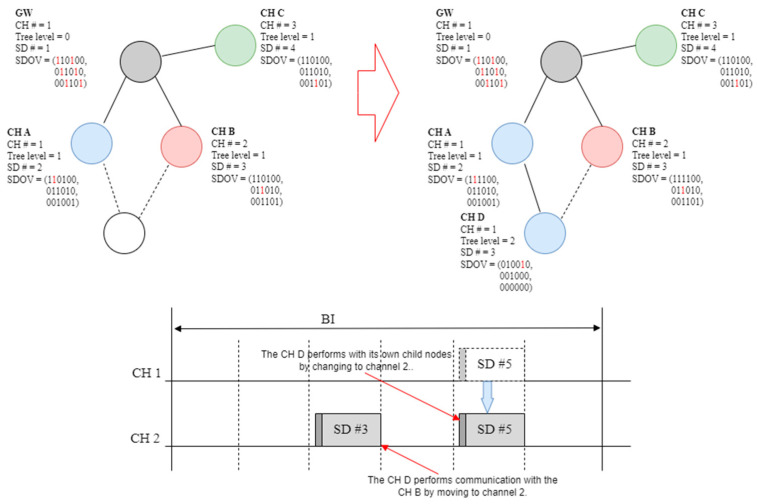
SD allocation algorithm to prevent collision during channel movement.

In Figure 18, in the proposed technique, when a new node receives a beacon frame from CH A and CH B, CH D can use one of the two radio channels as a primary channel and the other as a secondary channel. The proposed technique uses one channel as the main channel to transmit data, and the other can transmit data from the auxiliary channel when a problem occurs in the main channel or when urgent data are transmitted. There are no problems with regard to which channel was used as the main channel in the proposed technique. However, in the case shown in Figure 18, if CH D operates in SD 3 of channel 1, it collides with the SD section of CH B when the auxiliary channel is used. Accordingly, CH D cannot use channel 2 as an auxiliary channel. Therefore, when determining the SD number of the child cluster head, the SD selection algorithm is used (see Figure 19).

In Figure 19, when CH D receives a beacon frame from CH A and CH B, the main channel is set to one, and the SD section selects the SD number that does not overlap with the SD of CH A or CH B. In this case, one can communicate with one’s child nodes and parent node CH A through the main channel, and with CH B through a secondary channel if it is infeasible to communicate through the primary channel during the operation. As shown in Figure 18, CH D can perform this interference prevention algorithm by selecting an SD number of five, which does not overlap with CH A or CH B.

## 5. Performance Evaluations

The NS-2 simulator (version 2.34) was used to evaluate the performance of the proposed schemes. The common simulation parameters are summarized in Table 2. In the simulations, the nodes in the network operated in the beacon-enabled mode and were distributed randomly. In the simulation, the *BO* was set to six, and the *SO* was set to three. The cluster created by the gateway contained 30 nodes. The energy model in [60] defines four radio modes: transmitting, receiving, idle, and sleeping. The energy consumption was calculated by multiplying the time spent on the radio in each state by the energy consumption in that mode. To evaluate the consumed energy, the energy model of CC2630 (which is a single chip, 2.4 GHz, IEEE 802.15.4-compliant RF transceiver [60]) was used. In the simulation, clusters from other gateways were operated sequentially to determine the effect of interference from nearby devices. Gateway 1 began operation at 0.0 s. On the other side, gateway 2 started its operation at 253.19 s, and gateway 3 started its operation at 749.13 s. The simulations were run for 1000 s.

Table 3 lists the types of traffic generated by the nodes in the simulation.

**Figure 20:**
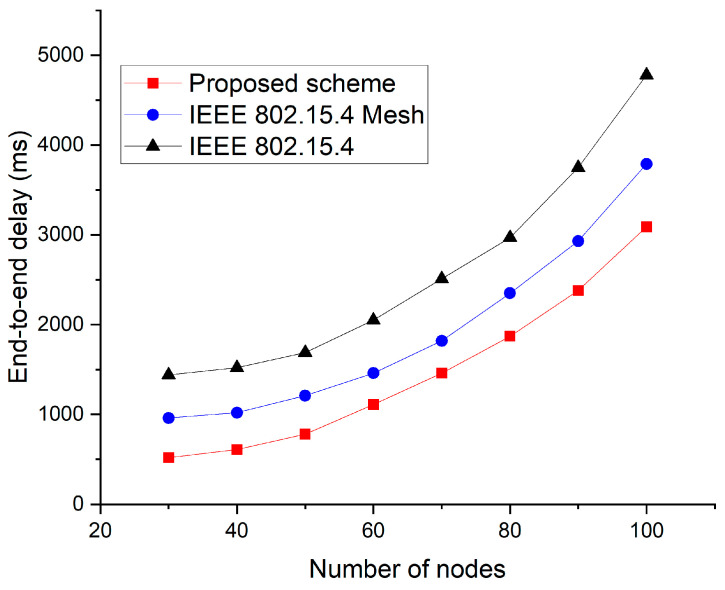
End-to-end delay vs. node number.

Figure 20 shows the end-to-end delay according to the increase in the number of nodes.

In this simulation, an arbitrary pair of nodes was selected to transmit data. The remaining nodes, except for the cluster head, generated data and transmitted these to the gateway. In Figure 20, the IEEE 802.15.4 Mesh protocol [53] shows a shorter latency than that of the IEEE 802.15.4 standard. This is because there are multiple paths for transmission to the destination node. In the proposed technique, data can be delivered through short paths because these do not pass through unnecessary gateways or cluster heads. In addition, the proposed method uses multiple channels. Hence, the number of competing nodes in the proposed technique is smaller than that in the other techniques. Therefore, the proposed technique has fewer collisions or backoffs and the shortest delay time.

**Figure 21:**
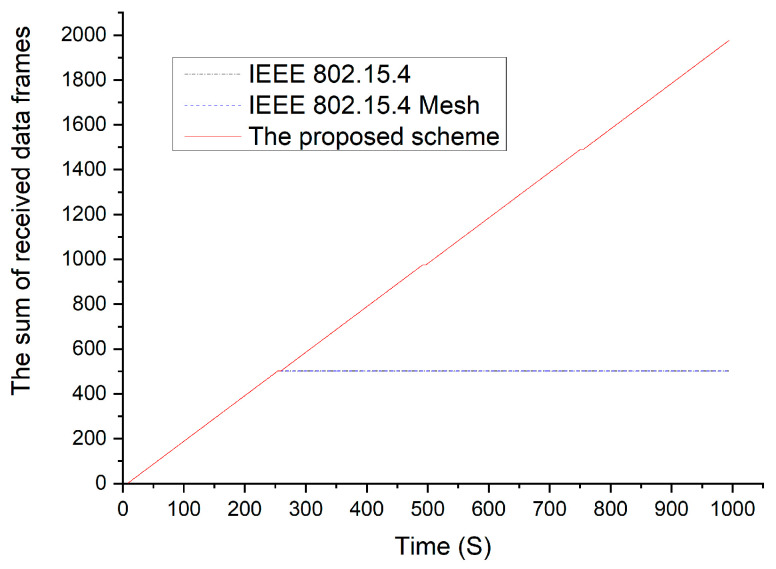
Number of received frames vs. time.

Figure 21 shows the sum of the received data frames with respect to time. In the IEEE 802.15.4 standard and IEEE 802.15.4 Mesh protocol, the sum of the received data frames did not increase because the device no longer received data after the data frames began to collide. However, the sum of the received data frames increased after a delay when the proposed scheme was used. In the proposed scheme, the gateway circumvents the use of resources because it can identify the channel and SD slot in use through the beacon frame received from a neighboring gateway. In addition, if a data frame conflict occurs between cluster members belonging to different gateways, the cluster head moves to the clean channel, selects the neighboring cluster head as the parent node, and notifies the gateway of the modification. This prevents data collisions.

**Figure 22:**
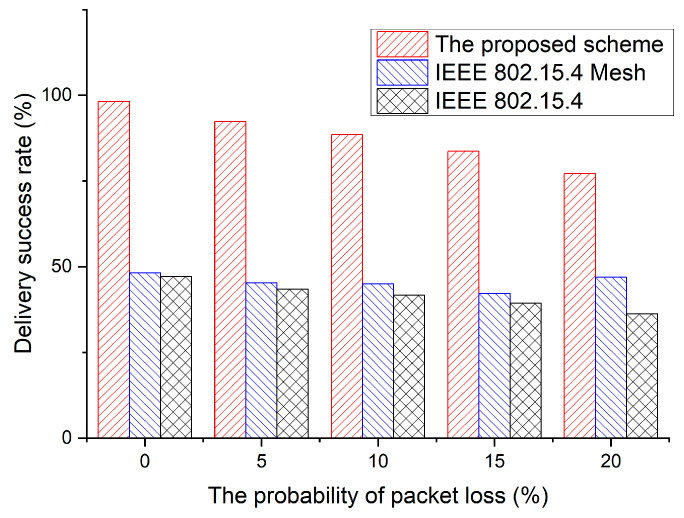
Comparison of the delivery success rate.

In Figure 22, the values of the delivery success rate are determined according to the probability of packet loss. To evaluate the performance of the proposed scheme, the delivery success rate was used. It is defined as follows:

$$R_{S}=\frac{N_{E}}{N_{C}}\times 100(\%)$$

where *N_E_* is the number of data frames received by the end device and *N_C_* is the number of data frames received by the coordinator device.

Packet loss is the ratio of packets dropped owing to interference to the total number of packets received. In this simulation, the delivery success rate of the proposed scheme was 98.19% when the packet loss was 0%, and 77.14% when the packet loss was 20%. When considering the beacon loss owing to transmission errors, a device adopting the proposed scheme loses 2.86% of the data frames. Meanwhile, the delivery success rate of the IEEE 802.15.4 standard and IEEE 802.15.4 mesh protocol was 47.13% when the packet loss was 0%. As the packet loss probability increases, the performance of the legacy protocol degrades significantly, and the difference in its performance from that of the proposed scheme increases further. This is because the IEEE 802.15.4 standard and IEEE 802.15.4 mesh protocol cannot prevent interference from other networks, and a larger interference causes more packet losses.

**Figure 23:**
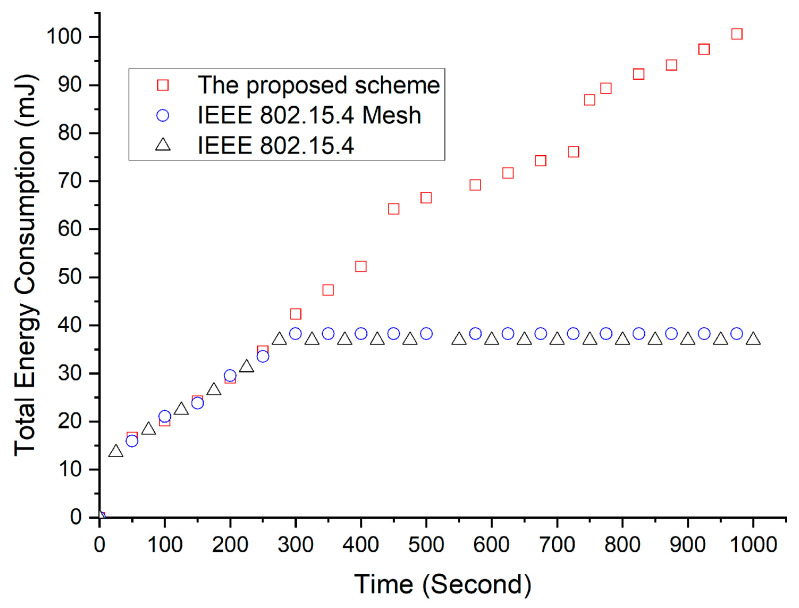
Energy consumption of a device that detects interference.

Figure 23 shows the dissipated power consumption of the device located in the overlapping region of the transmission ranges. The dissipated power consumption of the device that used the proposed scheme was equal to that of both a device that used the IEEE 802.15.4 standard and one that used the IEEE 802.15.4 Mesh protocol before data frames collide, as observed in this figure. At 253.19 s, the beacon frames first collided. Then, the devices that used the IEEE 802.15.4 standard and IEEE 802.15.4 mesh protocol performed an orphan scan after 253.19 s. In the simulation, the device that used the IEEE 802.15.4 standard and IEEE 802.15.4 mesh protocol attempted to synchronize with its cluster head. However, this device could not receive beacon frames and failed to synchronize because of beacon frame collisions. This legacy device restarted and attempted to associate with the cluster head but failed again because of beacon frame collisions. Finally, the device was switched off. Hence, the device using the legacy protocol did not consume energy after 270.58886 s.

**Figure 24:**
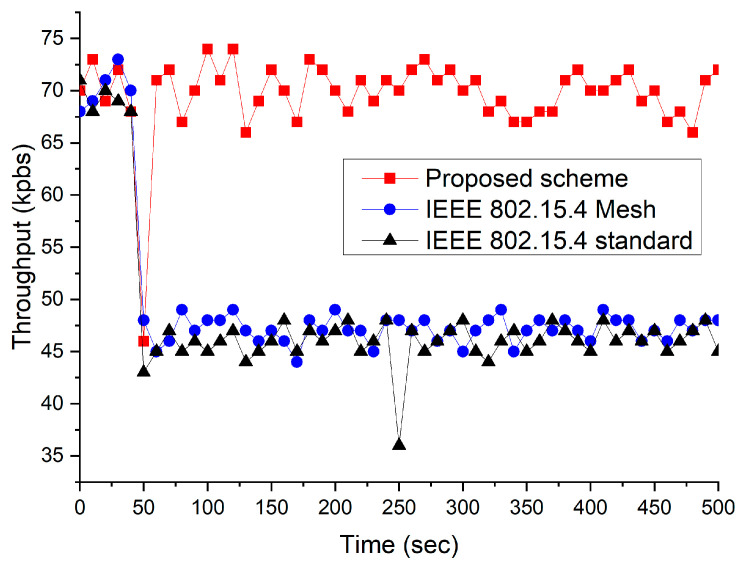
The throughput comparison when WLAN traffic load is low.

**Figure 25:**
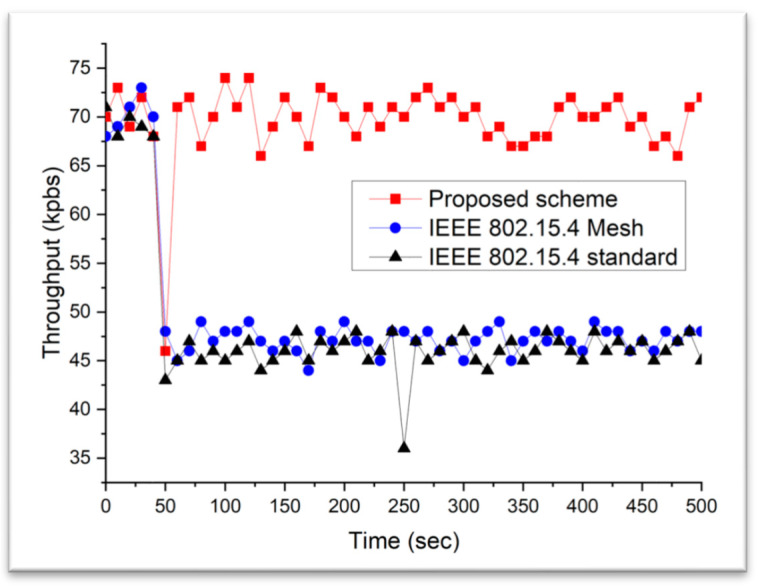
The throughput comparison when WLAN traffic load is high.

The next scenario considered a network environment with the presence of WLAN. Figure 24 and Figure 25 show the throughput of the interfered network when the traffic loads of the WLAN were 2 and 8 Mbps, respectively. In the simulation, the WLAN device was switched on for 50 s. It can be observed that the IEEE 802.15.4 standard and IEEE 802.15.4 mesh protocols were highly susceptible to the presence of the interferer. The network performance of the two protocols was degraded severely by the presence of the interferer because these do not provide an interference avoidance scheme. However, the proposed scheme could maintain the network performance, regardless of the presence of an interferer. This is because it can modify its network channel into an idle channel when it detects interference. Additionally, the proposed scheme provides a significantly faster recovery time. This is primarily because it can rapidly prevent channel interference by migrating the network channel to a predetermined idle channel.

**Figure 26:**
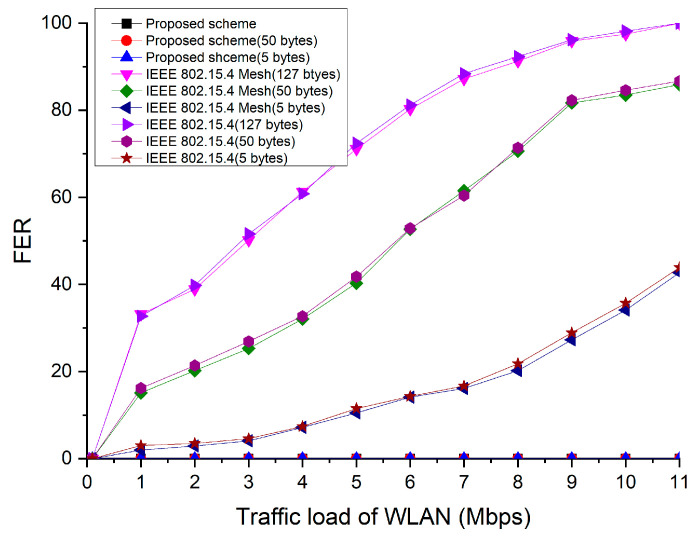
PER as a function of WLAN traffic load for different frame sizes.

Figure 26 depicts the packet error rate (PER) for different frame sizes of the IEEE 802.15.4 protocol. As the traffic load of the WLAN and size of the frame increased, the PER of the IEEE 802.15.4 standard and IEEE 802.15.4 mesh protocols increased significantly because the WLAN device could not circumvent interference. However, the PER of the proposed scheme did not vary regardless of the WLAN traffic load. This is because it can prevent interference by the WLAN.

**Figure 27:**
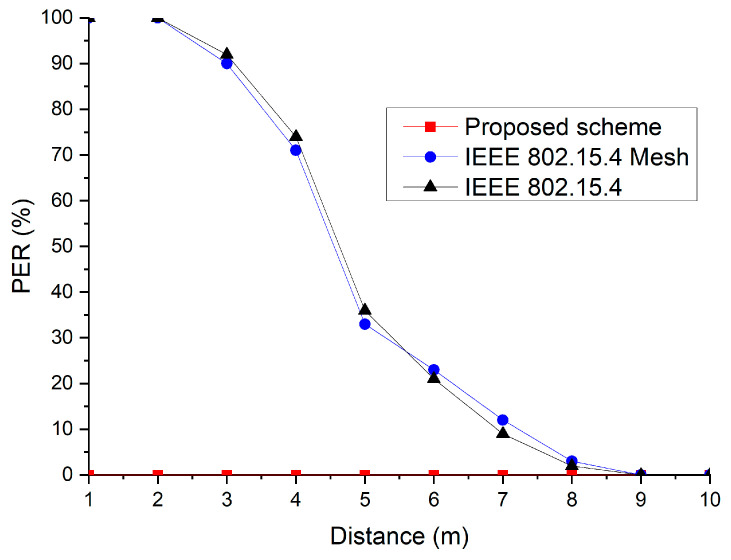
PER as a function of the distance between the IEEE 802.15.4 and WLAN devices.

Figure 27 shows the PER of the IEEE 802.15.4 network protocols under the interference of the WLAN with a 2 MHz center frequency offset. The distance between the IEEE 802.15.4 device and WLAN device varied from 0 m to 10 m. As the distance increased, the PER of the IEEE 802.15.4 standard and IEEE 802.15.4 mesh protocols decreased. This was because the power of the WLAN was concentrated at the center frequency. However, the proposed scheme maintained a constant PER regardless of this distance because it could prevent interference by the WLAN device.

## 6. Conclusions

In this study, a multi-channel cluster tree protocol was proposed for interference in time-critical smart-grid applications. To increase the network scalability in the typical IEEE 802.15.4 standard for WSNs, a network configuration method of a cluster tree topology was used. Moreover, a beacon enabled a mode with a superframe structure in which the active and inactive sections were repeated to reduce the power consumption. The proposed scheme can improve performance and reliability by proposing a channel allocation method for multi-channel operations and an intercluster scheduling method that prevents beacon collisions. The simulation results show how the network performance improves, compared with existing protocols, when the proposed scheme is used. To achieve better network load balancing, we plan to extend the proposed scheme using mechanisms such as data fusion or aggregation. This will enable us to further increase the speed of certain parts of the network in stable situations. Additionally, we intend to implement the proposed scheme in a real-world scenario testbed.

## Figures and Tables

**Table 1:** The bit string of each CH Info N field.

SD #	1	2	3	4	5	6	7	8
CH #								
1	1	0	0	0	0	0	0	0
2	0	1	0	0	0	0	0	0
3	0	0	1	0	0	0	0	0
4	0	0	0	1	0	0	0	0
5	0	0	0	0	1	0	0	0
6	0	0	0	0	0	1	0	0
7	0	0	0	0	0	0	1	0
8	0	0	0	0	0	0	0	1
9	1	0	0	0	0	0	0	0
10	0	1	0	0	0	0	0	0
11	0	0	1	0	0	0	0	0
12	0	0	0	1	0	0	0	0
13	0	0	0	0	1	0	0	0
14	0	0	0	0	0	1	0	0
15	0	0	0	0	0	0	1	0
16	0	0	0	0	0	0	0	1

**Table 2:** Simulation parameters.

Parameters	Value
Transmission power (dBm)	3.5
Noise factor (dB)	10.0
Synchronization mode	Beacon-enabled
Carrier sense sensitivity	−85 dBm
Data rate	250 kbps
IEEE 802.15.4 header length	22 bytes
RX current consumption	5.9 mA
TX current consumption	9.1 mA
IDLE current consumption	0.550 mA
Sleep current consumption	0.001 mA
*BO*	6
*SO*	3

**Table 3:** Smart grid application.

Traffic Type	Packet Interval	Packet Size
AMI data	18 s	5 bytes
SCADA (Supervisory Control and Data Acquisition)	1 s	64 bytes
Power Quality Monitoring	1 s	35 bytes
Office Substation	10 s	64 bytes

## Data Availability

The data used in this paper can be obtained by contacting the first author.
